# MITD1 Deficiency Suppresses Clear Cell Renal Cell Carcinoma Growth and Migration by Inducing Ferroptosis through the TAZ/SLC7A11 Pathway

**DOI:** 10.1155/2022/7560569

**Published:** 2022-08-22

**Authors:** Ye Zhang, Yanze Li, Qiangmin Qiu, Zhiyuan Chen, Yang Du, Xiuheng Liu

**Affiliations:** Department of Urology, Renmin Hospital of Wuhan University, Wuhan, 430060 Hubei, China

## Abstract

Clear cell renal cell carcinoma (ccRCC), the major histopathological subtype of renal cancer, is sensitive to ferroptosis. MIT-domain containing protein 1 (MITD1) has been reported to play an important role in hepatocellular carcinoma, while it remains unclear whether MITD1 is involved in ccRCC. Based on available data in The Cancer Genome Atlas, we found the expression of MITD1 increased through bioinformatics analysis and high MITD1 expression suggests a poor prognosis. And we validated that MITD1 expressed significantly in ccRCC through Western blot analysis. Then, we further compared the proliferation and migration capacity of ccRCC before and after MITD1 knockdown and further explored the effect of MITD1 knockdown on ferroptosis. The results indicated that MITD1 knockdown inhibited ccRCC cell proliferation and migration and induced ferroptosis in ccRCC. Furthermore, we found and analyzed the key molecule TAZ which was involved in ferroptosis caused by MITD1 knockdown. Subsequent overexpression experiments demonstrated that MITD1 knockdown induced ferroptosis and suppressed tumor growth and migration through the TAZ/SLC7A11 pathway. In summary, our study revealed the role of MITD1 in the ferroptosis of ccRCC and provided a novel target for ccRCC treatment.

## 1. Introduction

Renal cell carcinoma (RCC) originates from renal tubular epithelial cells and is one of the most common malignant tumors of the urinary system, of which the incidence is still increasing year by year [1]. Clear cell renal cell carcinoma (ccRCC) is the most common and aggressive type of RCC, which accounts for approximately more than 85% of RCC [2]. For the lack of clinical symptoms in the early stage of ccRCC, a considerable number of patients are diagnosed with locally advanced and even have distant metastases at the first-time consultancy. Localized ccRCC is generally cured by surgery, whereas patients with advanced ccRCC still have a poor prognosis due to the inability to undergo radical surgery [3]. Despite a variety of treatments including radiotherapy, chemotherapy, vascular endothelial growth factor (VEGF) receptor tyrosine kinase inhibitors, and immune-checkpoint inhibitors to delay the progress of the disease, it is hard to select treatment strategies for patients with advanced cancer due to the frequently occurring severe side effects and intrinsic or acquired drug resistance of each treatment [4, 5]. Therefore, it is necessary to further explore the mechanism of occurrence and development of ccRCC and to find more novel diagnostic markers and therapeutic targets.

MITD1 encodes MIT-domain containing protein 1 and participates in the process of cell division in the form of ESCRT-III dependence. MITD1 is able to be recruited to the midbody through the intermediate of MIT-domain of the N-terminus and ESCRT-III and coordinates abscission with earlier stages of cytokinesis [6, 7]. A study [8] found that MITD1 was able to serve as a predictor for human hepatocellular carcinoma prognosis and correlated with immune infiltrating cells around the carcinoma. In another research [9], the researchers identified MITD1 as one of the most important survival-related genes in bladder cancer, which was able to influence the migration ability of tumor cells by knocking down or overexpressing it. Despite some studies having found an abnormal expression of MITD1 in ccRCC, the role of MITD1 in the progression of ccRCC remains largely unknown.

Ferroptosis is a novel nonapoptotic form of death characterized by accumulation of intracellular reactive oxygen species (ROS), depletion of reduced glutathione (GSH), resulting in iron dependent accumulation of lipid hydroperoxides reaching cell-lethal levels [10]. It is generally accepted that ferroptosis is involved in a variety of physiological and pathological processes, including cancer, ischemia reperfusion injury, and neurodegeneration [11]. At present, ferroptosis has been considered to be closely related to tumor progression, indicating a potential means of cancer therapy [12]. Solute carrier family 7 membrane 11 (SLC7A11) is the major subunit of the cystine/glutamate antiporter (System Xc^−^), which is the key enzyme in the synthesis of GSH and resistance to ferroptosis [13]. Li et al. [14] found that inhibition of SLC7A11 induced ferroptosis in renal cancer. Therefore, ferroptosis may be a novel strategy for the treatment of RCC, and SLC7A11 is likely to be an extremely vital target.

In this study, we conducted a systematic bioinformatics analysis and found that MITD1 expression was significantly increased in ccRCC, of which the difference suggested different clinical prognoses. Further studies have shown that MITD1 knockdown of RCC cell lines with high expression of MITD1 decreased cell proliferation and migration ability. Notably, it was first observed that knockdown of MITD1 induced ferroptosis in ccRCC. Moreover, we confirmed that knockdown of MITD1 was able to regulate TAZ and reduce the expression of SLC7A11 to induce ferroptosis and decrease cell proliferation and migration ability. Our study provides a detailed analysis of the relationship between MITD1 and ferroptosis in ccRCC, which will aid in the treatment of ccRCC.

## 2. Materials and Methods

### 2.1. Bioinformatics Analysis

The expression of the target gene MITD1 in various malignant tumors was analyzed in the GEPIA (http://gepia.cancer-pku.cn/), a web-based tool to deliver fast and customizable functionalities based on TCGA and GTEx data. We utilized the TCGA (https://cancergenome.nih.gov/) database to obtain RCC data, containing all data of 611 ccRCC samples. We retained both primary RNA sequencing (RNA-seq) data and corresponding clinical information, which we used for comprehensive analysis of MITD1.

### 2.2. Cell Lines and Cell Culture

All cell lines were acquired from ATCC. HK-2 cell line was cultured with Dulbecco's modified eagle medium/10% fetal bovine serum (A3160901, Gibco) media while RCC cell lines (786-O, ACHN, A498, 769-P, Caki-1) were cultured with RPMI 1640/10% fetal bovine serum media. All cells were cultured in an incubator with 5% CO_2_ at 37°C.

### 2.3. Transfection

MITD1 siRNA, negative control siRNA, SLC7A11 plasmid, TAZ plasmid, and vector plasmid were synthesized by Sangon Biotech (Shanghai). RCC cells were seeded in 6-well plates at an appropriate density. After 12 h, transfection was then carried out using Lipofectamine 3000 (L3000001, Thermo Fisher Scientific) according to the manufacturer's instructions.

### 2.4. Cell Counting Kit-8-Based Cell Viability Assay

RCC cells were first seeded into 96-well plates at the density of 2 × 10^3^ cells/well. 10 *μ*L of CCK-8 solution (C0037, Beyotime Biotechnology) was added to each well and incubated for 1 h; the absorbance measurements at 450 nm were determined at 24 h, 48 h, 72 h, and 96 h.

### 2.5. Tumor Cell Colony Formation Assay

RCC cells were seeded into 6-well plates at approximately 200 cells per well and cultured for 14 days. Before counting, cells were fixed with 3.7% paraformaldehyde for 15 min and then stained with 0.1% crystal violet for 30 min. Colonies with >0.05 mm diameter were recorded and analyzed.

### 2.6. Wound Healing Assay

RCC cells were inoculated into 6-well plates at an appropriate density. When the cell density reached 90% to 95%, the surface of cells was scratched with a straight gap. Washed 3 times with phosphate buffer saline (PBS), the width of the gap was recorded 24 h later.

### 2.7. Western Blot Analysis

HK-2 cells and RCC cells were homogenized in RIPA lysis buffer containing protease inhibitors. We measured the protein concentration of each sample by bicinchoninic acid (BCA) assay and then were separated by SDS-PAGE. Transferred onto a piece of polyvinylidene difluoride transfer membrane, proteins of each sample were blocked with 5% nonfat milk for 1 h. The membranes were incubated with antibodies against MITD1 (PA5-116854, Cell Signaling Technology), SLC7A11 (ab37185, Abcam), glutathione peroxidase 4 (GPX4; ab125066, Abcam), cyclooxygenase 2 (COX2; ab62331, Abcam), acyl-CoA synthetase long-chain family member 4 (ACSL4; ab155282, Abcam), and glyceraldehyde 3-phosphate dehydrogenase (GAPDH; ab8245, Abcam). After incubation with primary antibodies for one night, the membranes were washed and incubated with secondary antibodies. The protein bands were visualized using enhanced chemiluminescence reagents (WP20005, Thermo Fisher Scientific). Finally, using ImageJ software performed the densitometric analysis to quantify differences in protein levels.

### 2.8. Measurement of ROS Level

After different treatments, RCC cells were incubated with 10 *μ*M dichlorodihydrofluorescein diacetate (DCFH-DA, S0033S-1, Beyotime Biotechnology) at 37°C for 20 min in the dark. Then, cell nuclei were labeled by using DAPI dihydrochloride (C1002, Beyotime Biotechnology) for 5 min. Finally, the cells were observed and photographed under a fluorescence microscope after washing with PBS three times.

### 2.9. Lipid Peroxidation Measurements

Malondialdehyde (MDA) is one of the end products of lipid peroxidation, which is widely accepted as a biomarker of lipid peroxidation. The level of MDA was detected by MDA assay kit (S0131S, Beyotime Biotechnology). After different treatments, the supernatant was collected and added to the assay kit. Then, the absorbance measurements at 450 nm were determined by a microplate reader.

### 2.10. Reduced Glutathione and Superoxide Dismutase

Reduced glutathione (GSH) and superoxide dismutase (SOD) were detected using Glutathione Detection Kit (S0053, Beyotime Biotechnology) and SOD assay kit (S0109, Beyotime Biotechnology) according to the manufacturer's instruction.

### 2.11. Statistical Analysis

All the data was expressed by mean value ± standard error and analyzed by SPSS 25.0. The differences between groups were analyzed through one-way analysis of variance (ANOVA) and the Student–Newman–Keuls test. *P* < 0 .05 was considered to be statistically significant.

## 3. Results

### 3.1. MITD1 Expression Is Upregulated in ccRCC Tumors and Correlated with the Progression and Prognosis

**Figure 1:**
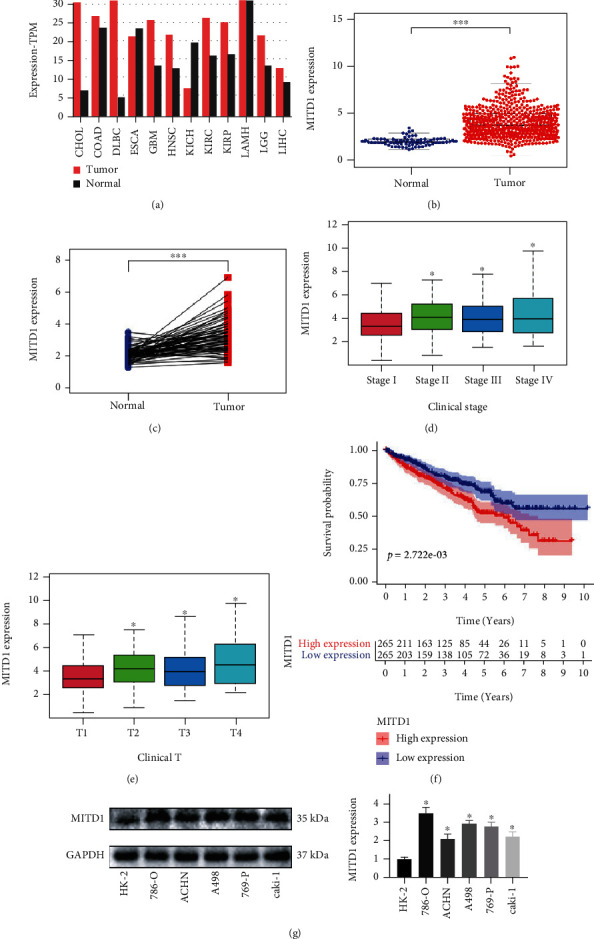
MITD1 was generally highly expressed in ccRRC. (a) Analysis from the GEPIA showing the expression of MITD1 in common malignant tumors. (b) The expression of MITD1 in ccRCC tissues was higher compared to the normal tissues through TCGA dataset analysis. (c) The expression of MITD1 in paired ccRCC tissues was also higher compared to the paracancerous tissues. (d, e) Relationship between MITD1 expression and tumor stage as well as that between MITD1 expression and T stage. (f) Based on the median values of MITD1 expression, patients were divided into the low-expression or high-expression group. Kaplan-Meier's survival curve of two groups through the analysis of clinical information of ccRCC in TCGA. (g) Representative blotting of MITD1 in different cell lines, and quantification of MITD1 proteins levels relative to HK-2 cells. Values are expressed as the mean ± SEM, *n* = 3. ^∗^*P* < 0.05 and ^∗∗∗^*P* < 0.001.

To assess the role of MITD1 in malignant tumors, we first investigated the expression of MITD1 in various malignant tumors in the GEPIA. As shown in Figure 1(a), MITD1 was generally highly expressed in tumors. Then, we further analyzed publicly available data of ccRCC from TCGA and found that the expression of MITD1 was significantly higher in tumor cases compared with normal cases in paired or unpaired ccRCC tissues (Figures 1(b) and 1(c)). What is more, the expression of MITD1 gradually increased with the increase of stage and depth of invasion (Figures 1(d) and 1(e)). And patients with high MITD1 expression had a lower overall survival rate than those with low expression (Figure 1(f)), suggesting that MITD1 was associated with the progression and prognosis. Finally, we verified that the expression of MITD1 in RCC cell lines was generally higher than that of HK2 cell lines and selected 786-O and A498 cell lines in subsequent experiments (Figure 1(g)).

### 3.2. MITD1 Knockdown Inhibits ccRCC Cell Proliferation and Migration

**Figure 2:**
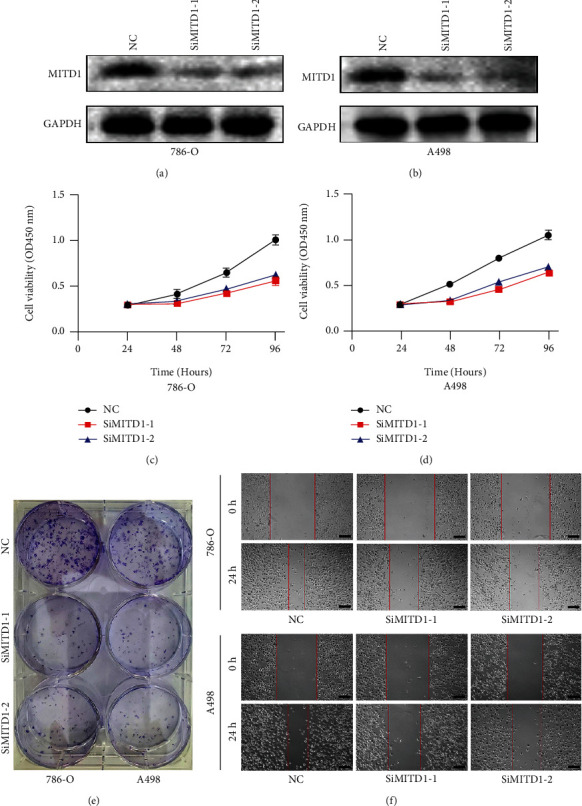
MITD1 knockdown inhibits ccRCC cell proliferation and migration. (a, b) 786-O cells or A498 cells were transfected with negative control or different si-RNA (SiMITD1-1 or SiMITD1-2). Western blot of MITD1 to test the effect of si-RNA transfection. (c, d) Knockdown of MITD1 suppressed cell growth ability in ccRCC cells (786-O and A498). (e) Proliferation of 786-O and A498 cells was suppressed by MITD1 knockdown. (f) When 786-O and A498 cells with different treatments grew to 90%-95% density, the surface of cells was scratched with a straight gap. Scratches at 0 and 24 hours were photographed and recorded under the microscope (magnification ×40; scale bars = 200 *μ*m). Values are expressed as the mean ± SEM, *n* = 3.

To explore the functional role of MITD1 in ccRCC, MITD1 siRNA was used to knock down the expression of MITD1. As shown in Figures 2(a) and 2(b), the expression of MITD1 in 786-O and A498 cells significantly reduced after MITD1 transient knockdown. CCK-8 and clone formation experiments indicated that MITD1 knockdown inhibited the proliferation (Figures 2(c) and 2(d)) and clonogenic capacity (Figure 2(e) and Supplementary Figure 1A) of ccRCC cells compared to the NC group. And the results of wound healing assays demonstrated that silencing of MITD1 prolonged the wound healing time, indicating that MITD1 knockdown was able to reduce the migration ability of ccRCC (Figure 2(f) and Supplementary Figure 1B).

### 3.3. MITD1 Deficiency Induces Ferroptosis in ccRCC

**Figure 3:**
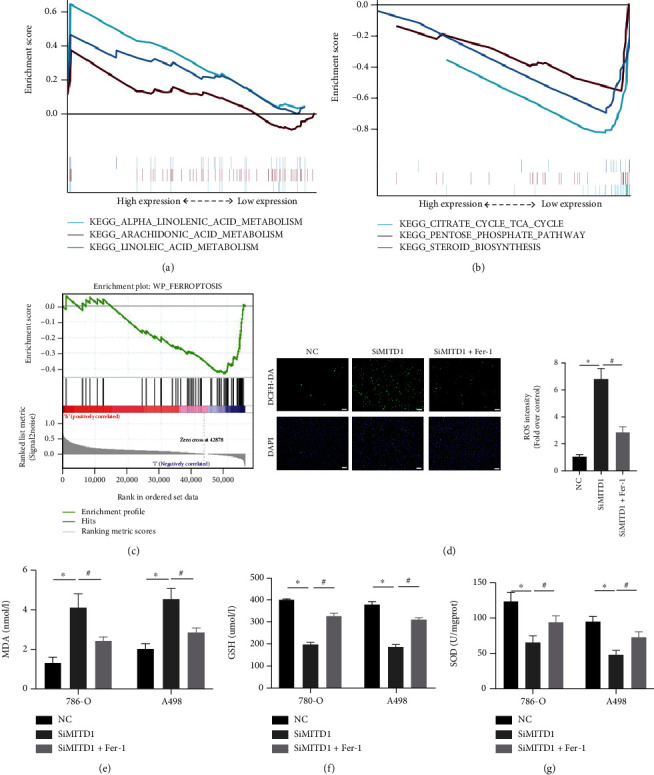
MITD1 knockdown induces ferroptosis in ccRCC. (a) KEGG pathway showed top three positively correlated groups. (b) KEGG pathway showed top three negatively correlated groups. (c) Results of MITD1 enriching to the ferroptosis pathway through GSEA platform with the WikiPathways. (d) Representative images of 786-O with DCFH-DA staining (magnification ×100; scale bars = 100 *μ*m) and their quantitative analysis. (d–f) Levels of MDA, GSH, and SOD in ccRCC cells with different treatments. Values are expressed as the mean ± SEM, *n* = 3. ^∗^*P* < 0.05, relative to the control group; ^#^*P* < 0.05, relative to the SiMITD1.

In order to determine how MITD1 depletion inhibits the proliferation and migration of ccRCC, KEGG pathway enrichment analyses were used to explore the potential pathway of MITD1. GSEA was used to reveal the significantly enriched (FDR < 0.05, *P* value < 0.05) KEGG pathways with high or low MITD1 expression. As shown in Figure 3(a), the top 3 KEGG pathways significantly correlated with MITD1 high expression were alpha-linolenic acid metabolism, arachidonic acid metabolism, and linoleic acid metabolism. And the top 3 pathways significantly correlated with MITD1 low expression were as follows: citrate cycle TCA cycle, pentose phosphate pathway, and steroid-biosynthesis (Figure 3(b)). In addition, we found that the remaining pathway significantly correlated with MITD1 low expression was not directly related to proliferation and migration. These results suggested that MITD1 might be associated with lipid and energy metabolism to regulate cell proliferation and migration. Therefore, we further explored whether MITD1 was involved in ferroptosis and found that MITD1 low expression was significantly associated with ferroptosis through the GSEA platform with the WikiPathways (c2.cp.wikipathways.v7.5.1.symbols.gmt) (Figure 3(c)). Subsequently, ROS levels of 786-O cells were detected by ROS assay kit.  Figure 3(d) showed that ROS levels obviously increased after knockdown of MITD1 while ROS levels of MITD1knockdown ccRCC cells recovered to some extent after Ferrostatin-1 (Fer-1, a potent ferroptosis inhibitor) treatment [15]. In addition, we assessed MDA, GSH, and SOD levels in ccRCC cells. As expected, MITD1 knockdown increased the level of MDA and reduced the level of antioxidant GSH and SOD in ccRCC cells; these changes were partially reversed by Fer-1 treatment (Figures 3(e)–3(g)). Moreover, we treated ccRCC cells with another potent inhibitor of ferroptosis (liproxstatin-1, Lip-1), and the results were consistent with Fer-1 treatment (Supplementary Figure 2), indicating that MITD1 was involved in ferroptosis.

### 3.4. MITD1 Deficiency Induces Ferroptosis through Downregulating SLC7A11

**Figure 4:**
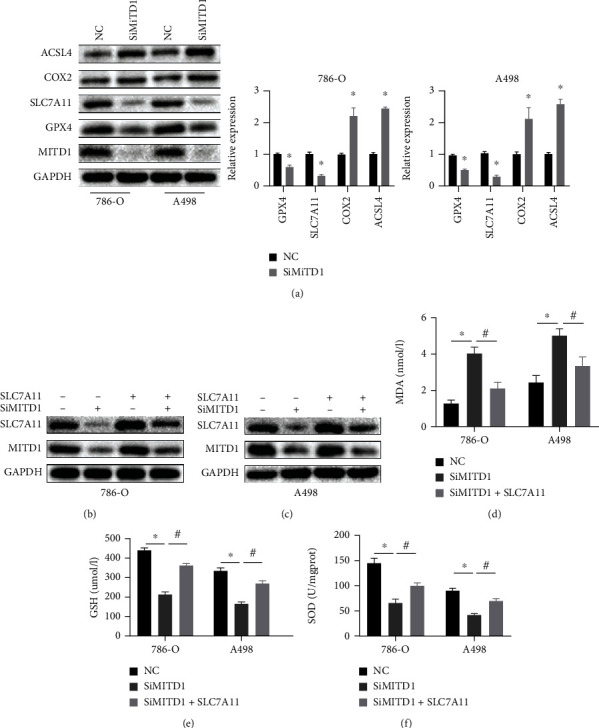
MITD1 knockdown induces ferroptosis through downregulating SLC7A11. (a) Western blot of MITD1and ferroptosis-related proteins (GPX4, SLC7A11, COX2, and ACSL4) in ccRCC cells after silencing MITD1 (SiMITD1). (b, c) 786-O cells or A498 cells were transfected with TAZ plasmid after silencing MITD1. Western blot of MITD1 and SLC7A11. (d–f) Levels of MDA, GSH, and SOD in ccRCC cells with different treatments. Values are expressed as the mean ± SEM, *n* = 3. ^∗^*P* < 0.05, relative to the control group; ^#^*P* < 0.05, relative to the SiMITD1.

To further confirm the impact of MITD1 on ferroptosis in ccRCC, ferroptosis-related proteins were detected in 786-O and A498 cells after MITD1 knockdown. As revealed in Figure 4(a), the expression of GPX4 and SLC7A11 was decreased and COX2 and ACSL4 were upregulated, further suggesting that silencing MITD1 induced ferroptosis. More specifically, the downregulation of SLC7A11 was the most significant, indicating that MITD1 deficiency might induce ferroptosis through SLC7A11. Subsequently, ccRCC cells were transfected with SLC7A11 plasmid or vector control after MITD1 knockdown or not. Figures 4(b) and 4(c) showed the expression level of MITD1 and SLC7A11 to demonstrate the effect of silence and overexpression in different ccRCC cells. We then examined the levels of MDA, GSH, and SOD in ccRCC with different treatments, which cloud reflect the degree of ferroptosis. Similarly, it was found that MDA increased significantly and GSH and SOD decreased significantly in ccRCC cells after MITD1 silencing. Furthermore, overexpression of SLC7A11 restored the levels of MDA, GSH, and SOD changed by MITD1 silencing (Figures 4(d)–4(f)), which demonstrated that MITD1 deficiency induced ferroptosis through SLC7A11.

### 3.5. MITD1 Knockdown Induces Ferroptosis through TAZ/SLC7A11 Pathway

**Figure 5:**
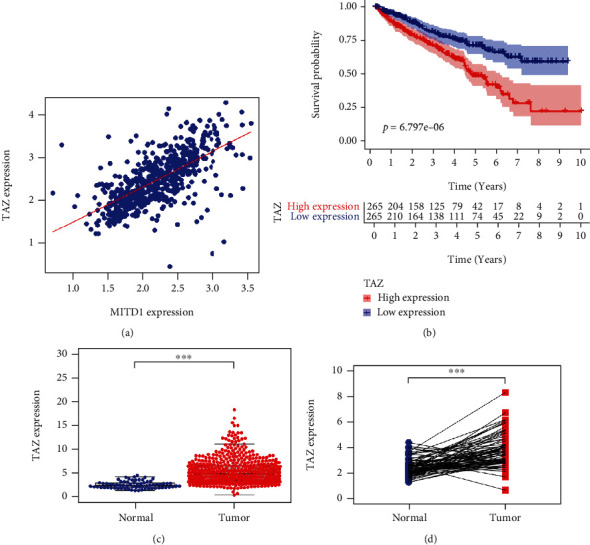
TAZ was highly correlated with MITD1 and high expressed in ccRRC. (a) A positive correlation between the MITD1 and TAZ expressions through bioinformatics analysis. (b) Based on the median values of TAZ expression, patients were divided into the low-expression or high-expression group. Kaplan-Meier's survival curve of two groups through the analysis of clinical information of ccRCC in TCGA. (c) The expression of TAZ in ccRCC tissues was higher compared to the normal tissues through TCGA dataset analysis. (d) The expression of TAZ in paired ccRCC tissues was also higher compared to the paracancerous tissues. Values are expressed as the mean ± SEM, *n* = 3. ^∗^*P* < 0.05 and ^∗∗∗^*P* < 0.001. relative to the control group.

To further investigate how MITD1 knockdown regulates ferroptosis in ccRCC, we screened coexpressed genes of MITD1 and discovered that the correlation between MITD1 and TAZ was extraordinarily strong (Figure 5(a)). And we further found that TAZ showed high expression in ccRCC and patients with high TAZ expression had a lower overall survival rate (Figures 5(b)–5(d)), which was consistent with MITD1. Furthermore, a recent study [16] found that YAP/TAZ induced the expression of SLC7A11 to enable HCC cells to overcome sorafenib-induced ferroptosis. Therefore, we proposed the idea that whether MITD1 knockdown promoted ferroptosis through regulating the TAZ/SLC7A11 pathway in ccRCC cells.

**Figure 6:**
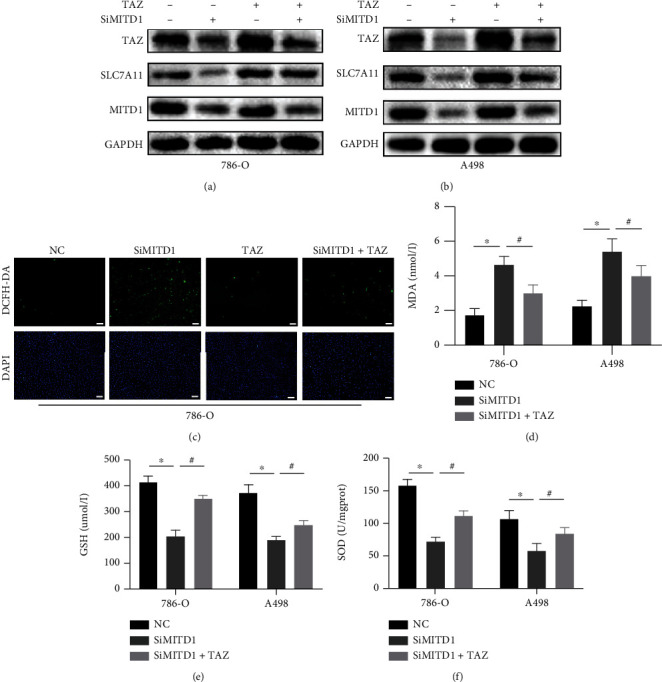
MITD1 knockdown induces ferroptosis through the TAZ/SLC7A11 pathway. (a, b) 786-O cells or A498 cells were transfected with negative control or si-RNA for MITD1 (SiMITD1) and then were transfected with TAZ plasmid. Western blot of MITD1, SLC7A11, and TAZ. (c) Representative images of 786-O cells with DCFH-DA staining (magnification ×100; scale bars = 100 *μ*m) after different treatments. (d–f) Levels of MDA, GSH, and SOD in ccRCC cells with different treatments. Values are expressed as the mean ± SEM, *n* = 3. ^∗^*P* < 0.05, relative to the control group; ^#^*P* < 0.05, relative to the SiMITD1.

In order to explore whether TAZ participated in the ferroptosis process induced by MITD1 knockdown, we carried out a series of experiments. In subsequent experiments, we found that the expression of TAZ was significantly reduced with downregulation of SLC7A11 after MITD1 knockdown in 786-O and A498 cells. What is more, overexpression of TAZ markedly increased the expression of SLC7A11 and also partially restored the down-regulation of SLC7A11 caused by MITD1 knockdown in different ccRCC cells (Figures 6(a) and 6(b)). Moreover, overexpression of TAZ reduced the levels of ROS which were obviously increased by MITD1 knockdown in ccRCC cells (Figure 6(c) and Supplementary Figure 1C). In addition, we also measured the level of MDA, GSH, and SOD. As shown in Figures 6(d)–6(f), MITD1 knockdown aggravated oxidative stress and ferroptosis which could be rescued by TAZ overexpression treatment.

### 3.6. MITD1 Knockdown Suppresses Growth and Migration through TAZ/SLC7A11 Pathway

**Figure 7:**
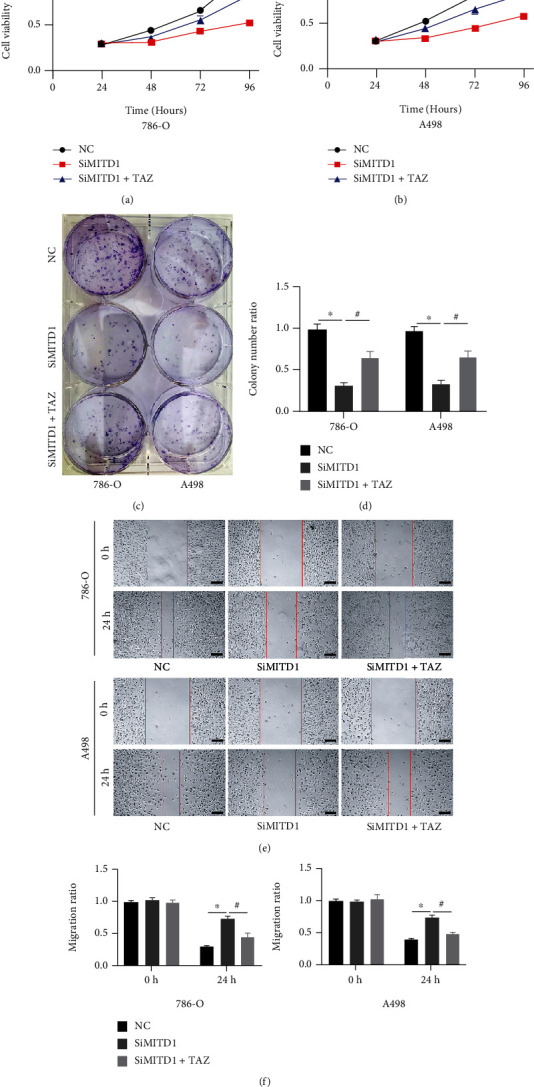
MITD1 knockdown suppresses growth and migration through the TAZ/SLC7A11 pathway. (a, b) 786-O cells or A498 cells were transfected with negative control or si-RNA for MITD1 (SiMITD1) and then were transfected with TAZ plasmid. Cell viability of each group was detected at 24 h, 48 h, 72 h, and 96 h, respectively. (c, d) Colony formation assay was performed on ccRCC cells with different treatments. The quantification data are also indicated. (e, f) When ccRCC cells with different treatments grew to 90%-95% density, the surface of cells was scratched with a straight gap. Scratches at 0 and 24 hours were photographed and recorded under the microscope (magnification ×40; scale bars = 200 *μ*m). The quantification data are also indicated. Values are expressed as the mean ± SEM, *n* = 3. ^∗^*P* < 0.05, relative to the control group; ^#^*P* < 0.05, relative to the SiMITD1.

To explore the role of SLC7A11 and TAZ expression changes caused by MITD1 knockdown in cell proliferation and migration, we performed a further series of experiments. The CCK-8 assay showed that the cell viability of the MITD1 knockdown group was significantly lower than that of the control group. After treatment with TAZ overexpression, the expression of TAZ and SLC7A11 increased and cell viability was partially restored (Figures 7(a) and 7(b)). Cell colony formation assay also demonstrated that TAZ overexpression rescued the reduction of cell proliferation caused by MITD1 knockdown (Figures 7(c) and 7(d)). Moreover, the wound-healing assay (Figures 7(e)abd 7(f)) showed that the change of cell migration ability caused by MITD1 knockdown could be rescued after TAZ overexpression treatment. These results indicated that MITD1 knockdown induced ferroptosis through downregulating SLC7A11and TAZ and inhibited the proliferation and migration ability of ccRCC. Therefore, MITD1 deficiency suppresses ccRCC growth and migration by inducing ferroptosis through the TAZ/SLC7A11 pathway.

## 4. Discussion

MITD1, an MIT-domain containing protein 1, recognizes subunits of ESCRT-III through a typical MIT-MIM1 readout in the dimer of MITD1. And the interaction of each other made the MITD1 recruit to the midbody during cell division [6]. Depletion of MITD1 is able to cause cytokinesis failure, which affects cell proliferation [17]. Previous studies [8, 9] revealed that MITD1 was abnormally expressed in patients with liver cancer and bladder cancer and had a significant correlation with prognosis. In addition, MITD1 regulated proliferation and invasion of tumor cells and was able to alter the tumor microenvironment by recruiting and regulating immune infiltrating cells [8, 18]. In our study, we analyzed public databases and found that MITD1 was highly expressed in ccRCC and the expression level was related to tumor stage and clinical T stage. In the subsequent survival analysis, patients in the MITD1 high expression group had a lower survival rate. In vitro experiment, it was verified that MITD1 was highly expressed in ccRCC through Western blot analysis of MITD1 expression in common RCC cell lines and HK2 cell lines. Furthermore, it was found that the growth, proliferation, and migration of ccRCC cells were inhibited after MITD1 knockdown treatment.

Ferroptosis has been identified as a nonapoptotic form of cell death, of which the two most important features are iron accumulation and lipid peroxidation. And lipid peroxidation has many manifestations, including an increase in ROS and a decrease in antioxidants [19]. Ferroptosis is involved in the development of many diseases, especially cancer [11, 20]. Therefore, inducing ferroptosis by destroying the redox balance in tumor cells may be an effective means of cancer treatment [21]. Although scholars have searched for key molecules that induce ferroptosis in ccRCC, related research is still rare. Wang et al. [22] found that SUV39H1 expression is frequently upregulated in ccRCC tumors and SUV39H1 knockdown induced iron accumulation and lipid peroxidation, leading to ferroptosis that disrupted ccRCC cell growth. Another study [23] showed that KLF2 inhibited the migration and invasion abilities of ccRCC cells by regulating ferroptosis through the GPX4 pathway. In this study, we found that MITD1was mainly related to the pathways of energy metabolism and lipid metabolism and could be enriched to the ferroptosis-related pathways through pathway enrichment analysis. We subsequently found that MITD1 deficiency increased ROS and MDA in ccRCC cells and decreased cellular GSH and SOD levels. However, the effects of MITD1 knockdown on these indicators were partially abolished by Fer-1 treatment. The above results demonstrated that MITD1 knockdown could induce ferroptosis in ccRCC.

The regulation of ferroptosis is a complex process that is affected by many factors, the two most important of which are transporter-dependent and enzyme-dependent [24]. System Xc^−^ is an amino acid transporter widely distributed on phospholipid bilayer membranes, mainly containing 2 subunits SLC7A11 and SLC3A2, which plays a key role in a transporter-dependent pathway [25]. System Xc^−^ is responsible for the transport of cystine into the cell and the transport of glutamate out of the cell. Cystine is reduced to cysteine after being transported into cells, which is the raw material for the synthesis of GSH. GSH is able to reduce intracellular ROS and lipid peroxidation through glutathione peroxidase (GPXs), the most typical of which is glutathione peroxidase 4 (GPX4) [25]. Inhibition of SLC7A11 reduces cystine absorption and glutathione synthesis, ultimately leading to oxidative damage and ferroptosis [26]. Therefore, targeting SLC7A11 is an important means to induce ferroptosis for cancer therapy. Inhibition of PARP downregulated the expression of SLC7A11 in a p53-dependent manner to promote ferroptosis in ovarian cancer cells, revealing a previously unrecognized mechanism of PARP inhibitor therapy for ovarian cancer [27]. Another study [14] in ccRCC found that SLC16A1-AS1 served as a sponge of miR-143-3p and knockdown of SLC16A1-AS1 could induce ferroptosis through the miR-143-3p/SLC7A11 pathway. In the present study, MITD1 deficiency could significantly affect the expression levels of ferroptosis proteins, such as GPX4, SLC7A11, ACSL4, and COX2. The downregulation of SLC7A11 was the most significant, indicating that MITD1 deficiency was most likely to induce ferroptosis by regulating SLC7A11. In addition, ROS, MDA, GSH, and SOD affected by MITD1 knockdown could be restored by overexpression of SLC7A11, further confirming the regulatory effect of MITD1 deficiency on SLC7A11.

Transcriptional coactivator with PDZ-binding motif (TAZ), which is also named as WW domain-containing transcriptional coregulatory 1 (WWTR1), is a key downstream effector of the Hippo signaling pathway [28]. Hippo signaling pathway is a potent tumor-suppressing mechanism and has been established as a novel determinant of ferroptosis [29]. Previous research [30] indicated that the expression of TAZ generally increased in kidney cancer tissue and cells and TAZ knockdown inhibited the proliferation, migration, and invasion of ccRCC in vitro and in vivo. Another study [31] found that the changes in cell density affect the expression of TAZ, which could regulate the sensitivity of renal cell carcinoma to ferroptosis through the EMP1-NOX4 pathway. Nevertheless, a latest study [16] demonstrated that YAP/TAZ could induce the expression of SLC7A11 to inhibit ferroptosis and maintain the resistance of liver cell carcinoma to sorafini. Based on correlation analysis, we found that MITD1 was highly correlated with TAZ. In addition, TAZ was highly expressed in ccRCC and TAZ high expression indicated a lower survival rate, same as MITD1. In the subsequent experiments, we found that MITD1 knockdown downregulated TAZ expression and a corresponding downregulation of SLC7A11 expression. In addition, overexpression of TAZ partially restored cellular ROS, MDA, GSH, and SOD changes caused by MITD1knockdown. Moreover, overexpression of TAZ also restored the proliferation and migration of ccRCC which were inhibited by MITD1 knockdown. These results suggested that MITD1 knockdown suppressed ccRCC growth and migration by inducing ferroptosis through the TAZ/SLC7A11 pathway.

## 5. Conclusion

In conclusion, our study firstly proposed that MITD1 could change the proliferative and migratory capacity of ccRCC and affect prognosis by regulating ferroptosis. What is more, we further explored that MITD1 deficiency could increase the ferroptosis of ccRCC through the TAZ/SLC7A11 pathway. Therefore, MITD1 is expected to be a prognostic biomarker of ccRCC and a new therapeutic target for tumor ferroptosis.

## Data Availability

Data supporting the findings of this study are included in the manuscript and the supplementary materials.

## Supplementary Materials

Supplementary Materials Supplemental Figure 1: quantified results for Figures 2(e) and 2(f) and DCFH-DA staining results of A498 cell line. Supplemental Figure 2: the results of DCFH-DA staining, SOD, GSH, and MDA treated with Lip-1 in ccRCC cells.

## References

[1] Siegel R. L., Miller K. D., Fuchs H. E., Jemal A. (2021). Cancer statistics, 2021. *CA: a Cancer Journal for Clinicians*.

[2] Ljungberg B., Bensalah K., Canfield S. (2015). EAU guidelines on renal cell carcinoma: 2014 update. *European Urology*.

[3] Capitanio U., Bensalah K., Bex A. (2019). Epidemiology of renal cell carcinoma. *European Urology*.

[4] Kotecha R. R., Motzer R. J., Voss M. H. (2019). Towards individualized therapy for metastatic renal cell carcinoma. *Nature Reviews Clinical Oncology*.

[5] Posadas E. M., Limvorasak S., Figlin R. A. (2017). Targeted therapies for renal cell carcinoma. *Nature Reviews Nephrology*.

[6] Hadders M. A., Agromayor M., Obita T. (2012). ESCRT-III binding protein MITD1 is involved in cytokinesis and has an unanticipated PLD fold that binds membranes. *Proceedings of the National Academy of Sciences of the United States of America*.

[7] Lee S., Chang J., Renvoise B., Tipirneni A., Yang S., Blackstone C. (2012). MITD1 is recruited to midbodies by ESCRT-III and participates in cytokinesis. *Molecular Biology of the Cell*.

[8] Shen H., Wang Z., Ren S. (2020). Prognostic biomarker MITD1 and its correlation with immune infiltrates in hepatocellular carcinoma (HCC). *International Immunopharmacology*.

[9] Chen Y., Xu T., Xie F. (2021). Evaluating the biological functions of the prognostic genes identified by the Pathology Atlas in bladder cancer. *Oncology Reports*.

[10] Li J., Cao F., Yin H. L. (2020). Ferroptosis: past, present and future. *Cell Death & Disease*.

[11] Jiang X., Stockwell B. R., Conrad M. (2021). Ferroptosis: mechanisms, biology and role in disease. *Nature Reviews. Molecular Cell Biology*.

[12] Dixon S. J. (2017). Ferroptosis: bug or feature?. *Immunological Reviews*.

[13] Koppula P., Zhang Y., Zhuang L., Gan B. (2018). Amino acid transporter SLC7A11/xCT at the crossroads of regulating redox homeostasis and nutrient dependency of cancer. *Cancer Communications*.

[14] Li Y. Z., Zhu H. C., Du Y., Zhao H. C., Wang L. (2022). Silencing lncRNA SLC16A1-AS1 induced ferroptosis in renal cell carcinoma through miR-143-3p/SLC7A11 signaling. *Technology in Cancer Research & Treatment*.

[15] Zilka O., Shah R., Li B. (2017). On the mechanism of cytoprotection by ferrostatin-1 and liproxstatin-1 and the role of lipid peroxidation in ferroptotic cell death. *ACS Central Science*.

[16] Gao R., Kalathur R., Coto-Llerena M. (2021). YAP/TAZ and ATF4 drive resistance to sorafenib in hepatocellular carcinoma by preventing ferroptosis. *EMBO Molecular Medicine*.

[17] Agromayor M., Martin-Serrano J. (2013). Knowing when to cut and run: mechanisms that control cytokinetic abscission. *Trends in Cell Biology*.

[18] Duan C. (2022). LncRNA SLC16A1-AS1 contributes to the progression of hepatocellular carcinoma cells by modulating miR-411/MITD1 axis. *Journal of Clinical Laboratory Analysis*.

[19] Li D., Li Y. (2020). The interaction between ferroptosis and lipid metabolism in cancer. *Signal Transduction and Targeted Therapy*.

[20] Tang D., Kroemer G. (2020). Ferroptosis. *Current Biology*.

[21] Hassannia B., Vandenabeele P., Vanden B. T. (2019). Targeting ferroptosis to iron out cancer. *Cancer Cell*.

[22] Wang J., Yin X., He W., Xue W., Zhang J., Huang Y. (2021). SUV39H1 deficiency suppresses clear cell renal cell carcinoma growth by inducing ferroptosis. *Acta Pharmaceutica Sinica B*.

[23] Lu Y., Qin H., Jiang B. (2021). KLF2 inhibits cancer cell migration and invasion by regulating ferroptosis through GPX4 in clear cell renal cell carcinoma. *Cancer Letters*.

[24] Yang W. S., Stockwell B. R. (2016). Ferroptosis: death by lipid peroxidation. *Trends in Cell Biology*.

[25] Liu M. R., Zhu W. T., Pei D. S. (2021). System Xc(-): a key regulatory target of ferroptosis in cancer. *Investigational New Drugs*.

[26] Lang X., Green M. D., Wang W. (2019). Radiotherapy and immunotherapy promote tumoral lipid oxidation and ferroptosis via synergistic repression of SLC7A11. *Cancer Discovery*.

[27] Hong T., Lei G., Chen X. (2021). PARP inhibition promotes ferroptosis via repressing SLC7A11 and synergizes with ferroptosis inducers in BRCA-proficient ovarian cancer. *Redox Biology*.

[28] Piccolo S., Dupont S., Cordenonsi M. (2014). The biology of YAP/TAZ: hippo signaling and beyond. *Physiological Reviews*.

[29] Sun T., Chi J. T. (2021). Regulation of ferroptosis in cancer cells by YAP/TAZ and Hippo pathways: the therapeutic implications. *Genes & Diseases*.

[30] Ruan H., Bao L., Song Z. (2019). High expression of TAZ serves as a novel prognostic biomarker and drives cancer progression in renal cancer. *Experimental Cell Research*.

[31] Yang W. H., Ding C. C., Sun T. (2019). The Hippo pathway effector TAZ regulates ferroptosis in renal cell carcinoma. *Cell Reports*.

