# Supplementary material for: MITD1 Deficiency Suppresses Clear Cell Renal Cell Carcinoma Growth and Migration by Inducing Ferroptosis through the TAZ/SLC7A11 Pathway
Source: Oxid Med Cell Longev. 2022 Aug 22;2022:7560569. doi: 10.1155/2022/7560569 (PMC9423985; doi:10.1155/2022/7560569)
Supplement: Supplementary Materials — Supplemental Figure 1: quantified results for Figures 2(e) and 2(f) and DCFH-DA staining results of A498 cell line. Supplemental Figure 2: the results of DCFH-DA staining, SOD, GSH, and MDA treated with Lip-1 in ccRCC cells. [file 7560569.f1.docx]

**Supplemental Materials**

**Supplemental Figure 1 Quantified results for Figure 2E-F and DCFH-DA staining results of A498 cell line.**


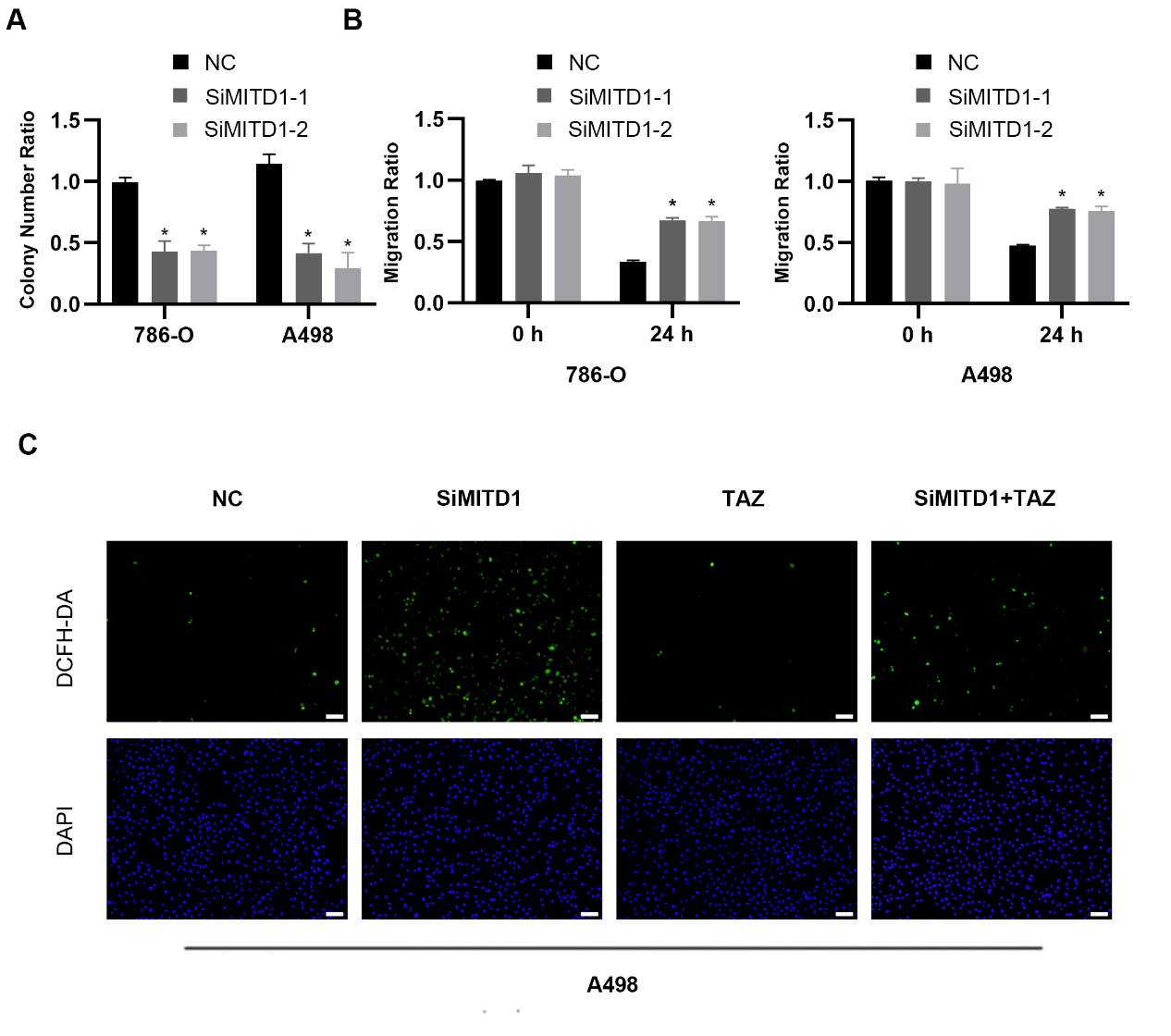


(A) 786-O cells or A498 cells were transfected with negative control or different si-RNA (SiMITD1-1 or SiMITD1-2). The quantification of colony formation assay of each group. (B) When 786-O and A498 cells with different treatments grew to 90%-95% density, the surface of cells was scratched with a straight gap. The quantification of scratch wound assay of each group at 0 and 24 hours. (C) 786-O cells or A498 cells were transfected with negative control or si-RNA for MITD1 (SiMITD1) and then were transfected with TAZ plasmid. Representative images of A498 cells with DCFH-DA staining (magnification ×100; scale bars = 100 μm) after different treatments. Values are expressed as the mean ± SEM, n=3. *P < 0.05, relative to control group.

**
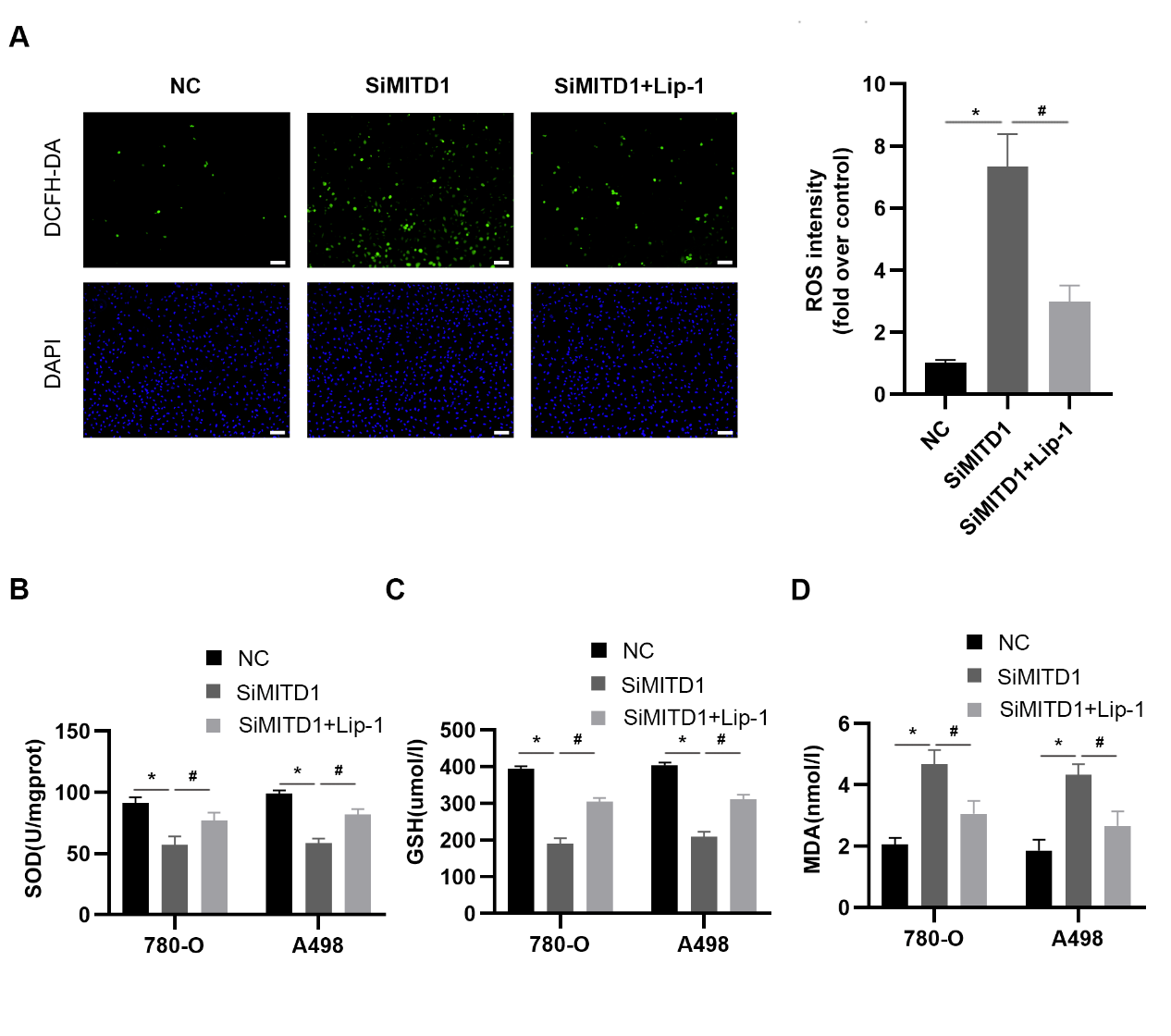
Supplemental Figure 2 The results of DCFH-DA staining, SOD, GSH, and MDA treated with Lip-1 in ccRCC cells.**

(A) ccRCC cells were transfected with negative control or si-RNA for MITD1 (SiMITD1) and then were treated with Lip-1. Representative images of 786-O with DCFH-DA staining (magnification ×100; scale bars = 100 μm) and their quantitative analysis (B-D) Levels of MDA, GSH and SOD in ccRCC cells with different treatments. Values are expressed as the mean ± SEM, n=3. *P < 0.05, relative to control group; #P < 0.05, relative to the SiMITD1.
